# Consideration for HPV vaccination recommendations in patients with vulvar dysplasia and malignancy following resection: A retrospective study investigating rural-urban vaccination practices in patients served by an academic medical center in the heartland

**DOI:** 10.1016/j.gore.2026.102067

**Published:** 2026-03-27

**Authors:** Zoe Keese, Kaeli Samson, Santiago Ortiz Barragan, Lindsey A. McAlarnen

**Affiliations:** a Department of Obstetrics and Gynecology, University of Nebraska Medical Center, Omaha, NE, USA; b Department of Biostatistics, College of Public Health, University of Nebraska Medical Center, Omaha, NE, USA; c Division of Gynecologic Oncology, Department of Obstetrics and Gynecology, University of Nebraska Medical Center, Fred and Pamela Buffett Cancer Center, 505 S. 45^th^ Street, Omaha, NE 68105, USA

**Keywords:** Vulva dysplasia, Vulvar cancer, HPV vaccination, Rural cancer prevention, Social vulnerability, adjuvant HPV vaccination

## Abstract

**Objectives:**

The American College of Obstetricians and Gynecologists (ACOG) recommends the HPV vaccine for unvaccinated or incompletely vaccinated individuals as an adjuvant treatment for cervical intraepithelial neoplasia (CIN) 2 + . Due to no such recommendation for adjuvant HPV vaccination for patients with vulvar intraepithelial neoplasia (VIN) 2 + after resection, and due to the large catchment area of our institution comprising rural and urban patient populations, which are disproportionately affected by HPV-associated cancers, we examined HPV vaccination uptake in patients with VIN2 + and vulvar malignancy and assessed relationships between social vulnerability and rurality.

**Methods:**

We performed a retrospective analysis of patients treated between 2018 and 2023 at a single Midwestern academic medical center. The study analyzed clinical and demographic data along with the associated social vulnerability index (SVI) and Rural-Urban Commuting Area (RUCA) codes.

**Results:**

Two hundred three unique patients met inclusion criteria. Of these, 36.5% (n = 74) lived in non-urban areas and 63.5% (n = 129) lived in urban areas. In the population of patients younger than 45 years of age who were not vaccinated for HPV prior to their VIN2 + diagnosis and treatment (n = 31), only 19.4% (n = 6) received at least one dose of the HPV vaccine in the adjuvant setting. Social vulnerability in housing and transportation was greatest in those living in rural areas.

**Conclusions:**

Extending the ACOG 2023 practice advisory recommendation for adjuvant HPV vaccination after excisional procedures for CIN2 + to encompass patients treated for VIN2 + may help reduce the burden of HPV-associated cancers.

## Highlights

• Adjuvant HPV vaccination recommendations for CIN2 + after excision do not extend to VIN2 + after excision.
• In the study population, social vulnerability in housing and transportation was greatest in those living in rural areas.
• Only 8.8% of eligible patients included had received a dose of the HPV vaccine prior to VIN2 + diagnosis.
• Including VIN2+ in adjuvant HPV vaccination recommendations may help reduce the burden of HPV-associated cancers.

## Graphical abstract

**Figure:**
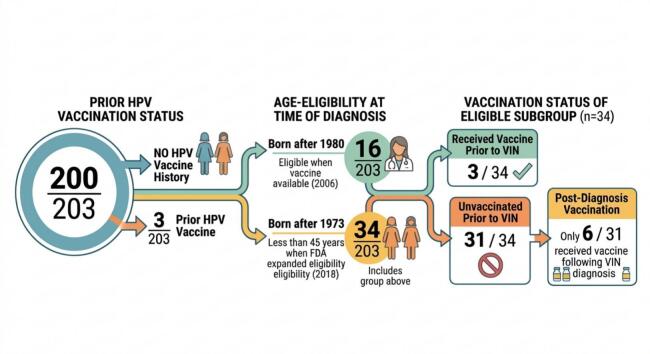

## 1 Introduction

Extensive evidence supports human papillomavirus (HPV) vaccination as a preventative measure. The percentage of cervical pre-cancers caused by HPV 16 and 18 dropped by 40 percent following the routine use of HPV vaccination in the United States (Centers for Disease Control and Prevention. Reasons to get vaccinated [Internet]. Atlanta (GA): CDC;, 2024). In recent years, however, the role of the HPV vaccine has extended beyond prevention, with emerging evidence that it can play an important role in adjuvant treatment for cervical intraepithelial neoplasia (CIN) 2+ (Lichter et al., 2020 May). Adjuvant HPV vaccination in women ages 15–45 undergoing treatment for cervical dysplasia is associated with a significantly reduced risk of recurrence of CIN2 + by 64% and CIN1 + by 33% in 6–48 months after treatment (Bartels et al., 2020). These findings have informed clinical recommendations. A 2023 American College of Obstetricians and Gynecologists (ACOG) practice advisory recommends that physicians treating previously unvaccinated patients aged 27–45 years with CIN2 + consider adjuvant HPV vaccination after excisional procedure to help prevent recurrence and progression of cervical dysplasia (American College of Obstetricians and Gynecologists, 2023 Jul, Sharpless et al., 2023).

Such a strategy and recommendation may be applied to vulvar dysplasia. HPV is detected in approximately 76% of vulvar dysplasia cases (Zhang et al., 2018 Sep 26) and at least 34–39% of invasive vulvar cancer cases (Ayala et al., 2023). Despite an ACOG practice advisory on adjuvant HPV vaccination in HPV-related cervical disease, there is a dearth of evidence and no recommendations for adjuvant HPV vaccination in vulvar intraepithelial neoplasia (VIN) 2 + and vulvar malignancy (Mix JM, Gopalani SV, Simko S, Saraiya M. Trends in HPV- and non-HPV-associated vulvar cancer incidence, United States, 2022, Ghelardi et al., 2021 Jan 25). There is great unmet potential for the HPV vaccination to decrease morbidity and mortality, though additional research is needed.

Rural patients face disproportionately worse outcomes for many cancers (Semprini et al., 2024 Aug 1). Rural populations have higher overall cancer incidence rates and higher rates of cancers with preventive opportunities compared with urban populations; improvements in these rates are typically slower in rural populations (Semprini et al., 2025). Further, the incidence of HPV-associated cancers is higher in rural areas compared to metropolitan areas, and this disparity has increased in recent years (Semprini et al., 2024 Aug 1). In an analysis of the North American Association of Central Cancer Registries data set, females in rural areas exhibited significantly higher rates of vulvar squamous cell carcinoma, vaginal squamous cell carcinoma, and cervical carcinoma compared to urban peers (Zahnd et al., 2019).

While cervical cancer has decreased in some populations due to the HPV vaccine, vaccination in rural areas has lagged behind urban populations (Semprini et al., 2024 Aug 1). Because of these trends, we examined HPV vaccination uptake in patients with VIN2 + and vulvar malignancy treated at our academic tertiary care center, which serves a large rural population in the heartland. We assessed relationships between social vulnerability and rurality in this population of patients with vulvar dysplasia and cancer.

## 2 Methods

**Fig. 1:**
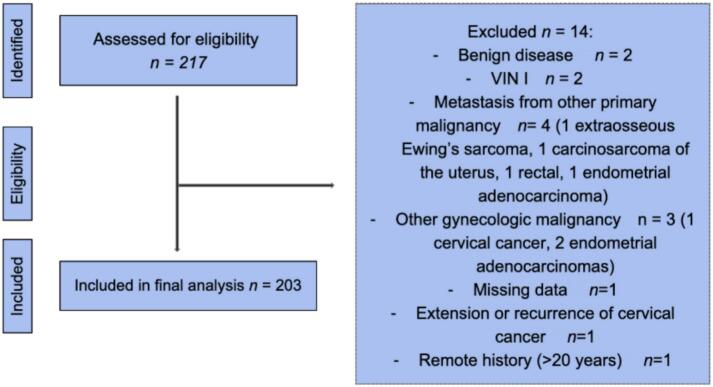
Consort diagram. Fourteen patients from the initial cohort of 217 were excluded for the following reasons: benign pathology (n = 2); VIN I (n = 2); metastasis from another primary malignancy (n = 4: extraosseous Ewing’s sarcoma, uterine carcinosarcoma, rectal primary, and endometrial adenocarcinoma); other gynecologic malignancy (n = 3: cervical cancer and two cases of endometrial adenocarcinoma); missing data (n = 1); extension or recurrence of cervical cancer (n = 1); and remote history > 20 years (n = 1).

We conducted an Institutional Review Board approved (IRB#00005662) retrospective analysis of patients diagnosed with vulvar dysplasia (VIN2 + ) or malignancy who received care at the University of Nebraska Medical Center. A waiver of informed consent was granted due to the retrospective design and use of existing medical records. This academic tertiary care institution serves the 93 counties of Nebraska and western Iowa. Ninety-one of 93 counties in Nebraska do not have a gynecologic oncology primary practice. Patients aged 19–99 years were included. Fourteen patients from the initial cohort of 217 were excluded (Fig. 1). Inclusion criteria were: age ≥ 19 years, receipt of care > 1 clinic visit at the University of Nebraska Medical Center between January 1, 2018 and December 31, 2023, and a diagnosis of vulvar intraepithelial neoplasia grade 2 or higher (VIN2 + ) or vulvar malignancy. Exclusion criteria included benign pathology, VIN1, diagnosis of a non-vulvar primary malignancy, metastatic disease to the vulva, missing significant key clinical data, and vulvar dysplasia treated outside the five years studied. Variables collected included HPV vaccination status, demographic characteristics, clinical stage, recurrence, and social vulnerability and rurality measures. The HPV vaccination status was taken from the electronic medical record immunization data, which integrates information from the hospital system and the Nebraska State Immunization Information System, a secure web-based system maintained by Nebraska Department of Health and Human Services. Data was verified by manual chart review.

Age was defined at the date of first surgery, or if no surgery occurred, at the date of first adjuvant treatment, or if neither occurred, at the date of first encounter. Because no adjuvant HPV vaccination recommendations currently exist for vulvar dysplasia or cancer, we extrapolated from the cervical CIN2 + ACOG practice advisory recommendation and FDA approval of the HPV vaccine age cutoff of ≤ 45 years to identify potentially eligible patients for adjuvant HPV vaccination following resection for VIN 2 + or malignancy.

Area-level measures of socioeconomic status, household composition and disability, minority status and language, and housing and transportation were assessed using the Centers for Disease Control and Prevention (CDC) 2022 Social Vulnerability Index (SVI). The SVI is a census tract-level composite measure derived from the U.S. Census Bureau and American Community Survey data. SVI scores are expressed as percentile rankings ranging from 0 to 1 with higher values indicating greater vulnerability. For analysis, SVI scores were categorized as low vulnerability (0.00–0.25), low–medium (0.2501–0.50), medium–high (0.5001–0.75), and high (0.7501–1.00).

Social vulnerability indices were analyzed alongside Rural-Urban Commuting Area (RUCA) codes, which were used to classify zip codes as urban, urban-adjacent, or non-adjacent. Urban included metropolitan core areas, metropolitan high commuting areas, and metropolitan low commuting areas (RUCA 1–3). Urban-adjacent included micropolitan core areas, micropolitan high commuting areas, small town core areas, and small town high commuting areas (RUCA 4,5,7,8). Non-adjacent rural included micropolitan low commuting areas, small town low commuting areas, and rural areas (RUCA 6,9,10).

Descriptive statistics were summarized as means ± SD or medians with IQRs. Categorical variables were compared using chi-square or Fisher’s exact tests. SVI differences were assessed using Spearman correlations, independent samples t-tests, Wilcoxon rank-sum, or Kruskal-Wallis tests (with Wilcoxon rank-sum Bonferroni-adjusted post hoc comparisons, if significant). All analyses were performed using SAS software version 9.4 (SAS Institute Inc., Cary, NC).

## 3 Results

In this study, 203 patients met inclusion criteria. The median age at encounter was 60.8 years (IQR 52.1–72.4). Table 1 presents demographic and clinical characteristics. The most common histologic subtypes were vulvar intraepithelial neoplasia (43.8% n = 89) and squamous cell carcinoma (42.4% n = 86). Regarding patients’ geographic groups, 63.5% (n = 129) were categorized as urban, 22.7% (n = 46) as urban-adjacent rural, and 13.8% (n = 28) as nonurban-adjacent rural.

**Table 1:** Patient Characteristics.

**Characteristic**	**n (%) or Median (IQR)**
Age (years) at encounter	60.8 (52.1–72.4)
**Insurance**	
None	3 (1.5)
Private	54 (26.6)
Public	146 (71.9)
**RUCA**	
Urban	129 (63.5)
Urban-adjacent rural	46 (22.7)
Nonurban-adjacent rural	28 (13.8)
**Race**	
Black or African American	14 (7.0)
Other	4 (2.0)
White or Caucasian	183 (91.0)
Unknown	2
**Histology**	
Adenocarcinoma	5 (2.5)
Basal cell carcinoma	5 (2.5)
Extramammary Paget’s	12 (5.9)
Malignant melanoma	3 (1.5)
Sarcoma	3 (1.5)
Squamous cell carcinoma	86 (42.4)
VIN 2+	89 (43.8)

Exploratory analyses did not reveal significant differences in SVI scores or RUCA classifications across subgroups defined by HPV vaccination status, stage at diagnosis, cancer development, or recurrence, though these analyses were likely underpowered to detect such differences. Vulnerability in housing and transportation was significantly higher in the urban-adjacent rural group relative to the urban group (p = 0.003), and vulnerability associated with minority status and language was highest in the urban group, relative to the urban-adjacent rural group (p = 0.04) and the nonurban-adjacent group (p < 0.001).

### 3.1 HPV vaccination status explored

Of patients analyzed, 98.5% (n = 200) had no history of HPV vaccination; only 1.5% (n = 3) had received at least one dose prior to diagnosis. Sixteen patients (7.9% of the total cohort) were born in or after 1980, and therefore would have been ≤ 26 years old when the HPV vaccine became available in 2006, making them historically vaccine eligible. Thirty-four patients (16.7% of the total cohort) were born in or after 1973, making them age < 45 in 2018 when the FDA extended eligibility to those ages 27–45.

Among all patients, during their disease course, 34 (16.7%) were 45 years old or younger—the upper age limit for FDA approval of the 9-valent HPV vaccine and the cutoff for eligibility to receive adjuvant HPV vaccination after CIN 2 + treatment if previously unvaccinated, per the 2023 ACOG practice advisory. Out of patients 45 years or younger (n = 34), 8.8% (n = 3) had a documented history of having the HPV vaccine. Of those in that same age group who were previously unvaccinated (n = 31), 19.4% (n = 6) received an HPV vaccine following diagnosis, including two patients with only one dose, one patient with only two doses, and three patients with three doses.

## 4 Discussion

This study highlights critically low HPV vaccination rates among patients diagnosed with vulvar dysplasia and vulvar malignancy in Nebraska and western Iowa. Out of the 31 patients who were age eligible for the HPV vaccine and not previously vaccinated for HPV, only 19.4% (n = 6) received HPV vaccination. Our extrapolation of cervical CIN2 + adjuvant vaccination recommendations underscores the current absence of vulvar-specific guidance from national medical societies and highlights the need for evidence-based recommendations for VIN2 + .

The study also includes a relatively large contemporary dataset of a rare condition with more than 1/3 of patients from non-metropolitan areas. Patients seen in both the benign gynecology practice and the cancer center were included in the analysis.

Identifying increased vulnerability in transportation and housing among rural patients in the population studied here is consistent with prior reports that rural communities face structural barriers limiting access to preventive care, including HPV vaccination (Semprini et al., 2024 Aug 1). This underscores the need to direct resources and clinical interventions toward rural patients with vulvar dysplasia receiving care at the tertiary center.

An additional barrier to adjuvant HPV vaccination is insurance coverage for vaccination in adults aged 27–45. While the FDA approval and ACOG support shared decision making for this age group, HPV vaccines are not universally covered as a standard preventive benefit under Medicare Part B. Coverage under Medicare Part D varies by plan and may require prior authorization in some plans. In Nebraska, Medicaid policies indicate that coverage for adults over age 26 may require justification or authorization. These insurance and reimbursement challenges amidst an ever-changing health policy climate highlight the need for clearer medical guideline-driven coverage policies to support adjuvant vaccination, particularly for populations more likely to use public insurance.

Based on the results of this study, our institution has taken steps to institute quality improvement initiatives to increase HPV vaccination, particularly following treatment for VIN2 + . These include HPV vaccination incorporation into dysplasia procedure order sets, educational materials for patients, and working to make the vaccination accessible at rural outreach clinics served by faculty. For those institutions that treat a large rural population, such quality improvement efforts are essential, but outcome data and policy assistance are needed to facilitate the clinical potential of widespread vaccination.

Current studies examining the use of the HPV vaccine in the treatment of women with VIN have reported reduced lesion size, symptom relief, histological regression, and HPV clearance, but no RCT results are available currently (Bryan et al., 2019 Jan 7). A phase IV trial, “HPV Vaccine to Interrupt Progression of Vulvar and Anal Neoplasia (VIVA),” was terminated early due to futility (Stankiewicz Karita et al., 2019 Apr 5). Results from these studies must help inform policy for vulvar dysplasia in addition to cervical dysplasia.

With the demonstrated benefit of HPV vaccination in cervical dysplasia and malignancy and the mechanistic similarities between usual-type VIN and CIN, there is a strong rationale to consider extending ACOG’s HPV vaccination adjuvant treatment recommendation for CIN2 + to VIN 2 + . Such an advisory could provide clearer guidance to clinicians, standardize care, and help promote insurance coverage for a vaccine that can provide a second chance at primary prevention for a condition disproportionally affecting patients living in rural areas. Our study has limitations, most notably > 80% were not eligible for the HPV vaccine as they were older than 45 when treated for vulvar dysplasia or cancer. Should they have received the vaccine prior to age 45, or best yet, at age 9–12, we suspect the incidence of vulvar dysplasia and cancer would be much lower, theorized to reduce the occurrence of usual-type VIN up to 80 to 90% when the 9-valent vaccine is administered on time (Sharpless et al., 2023). Additionally, data is retrospective, from a single institution, and may not be generalizable to all institutions with large catchment areas treating rural populations.

Recently, five national societies, including the Society of Gynecologic Oncology and the American College of Obstetricians and Gynecologists, published consensus recommendations on health equity, disparities, and barriers to cervical cancer care in the US addressing WHO Cervical Cancer Elimination Campaign goals, noting that the factor of rurality impacts access to services, is associated with increased late-stage presentation, and contributes to lower rates of completion of curative-intent treatment for cervical cancer (Neibart et al., 2025). Multi-level policy reform is cited as the “greatest hope” for expeditious change. In conjunction with widespread cervical cancer elimination efforts, we advocate for the inclusion of vulvar dysplasia and cancer in such efforts. In particular, inclusion of VIN2 + in adjuvant HPV vaccination recommendations would allow all patients with HPV-associated vulvar dysplasia and cancers to equitably access life-changing treatment.

## CRediT authorship contribution statement

**Zoe Keese:** Writing – review & editing, Writing – original draft, Data curation, Conceptualization. **Kaeli Samson:** Writing – review & editing, Validation, Formal analysis, Data curation. **Santiago Ortiz Barragan:** Writing – review & editing, Writing – original draft, Project administration, Data curation. **Lindsey A. McAlarnen:** Writing – review & editing, Writing – original draft, Supervision, Project administration, Funding acquisition, Data curation, Conceptualization.

## Declaration of competing interest

The authors declare that they have no known competing financial interests or personal relationships that could have appeared to influence the work reported in this paper.
